# Improved lysis efficiency and immunogenicity of *Salmonella* ghosts mediated by co-expression of λ phage holin-endolysin and ɸX174 gene *E*

**DOI:** 10.1038/srep45139

**Published:** 2017-03-23

**Authors:** Gayeon Won, Irshad Ahmed Hajam, John Hwa Lee

**Affiliations:** 1 College of Veterinary Medicine and Bio-Safety Research Institute, Chonbuk National University, Iksan campus, Gobong-ro-79 Iksan 54596, South Korea.

## Abstract

Bacterial ghosts (BGs) are empty cell envelopes derived from Gram-negative bacteria by bacteriophage ɸX174 gene *E* mediated lysis. They represent a novel inactivated vaccine platform; however, the practical application of BGs for human vaccines seems to be limited due to the safety concerns on the presence of viable cells in BGs. Therefore, to improve the lysis efficiency of the gene *E*, we exploited the peptidoglycan hydrolyzing ability of the *λ* phage holin-endolysins to expedite the process of current BG production system. In this report, we constructed a novel ghost plasmid encoding protein E and holin-endolysins in tandem. We observed that sequential expressions of the gene *E* and the holin-endolysins elicited rapid and highly efficient *Salmonella* lysis compared to the lysis mediated by gene *E* only. These lysed BGs displayed improved immunogenicity in mice compared to the gene *E* mediated BGs. Consequently, seventy percent of the mice immunized with these novel ghosts survived against a lethal challenge while all the mice vaccinated with gene *E* mediated ghosts died by day 9 post-infection. We conclude that this novel strategy has the potential to generate highly efficient inactivated candidate vaccines that could replace the currently available bacterial vaccines.

Lytic bacteriophages induce bacterial cell lysis to release progeny virions from their host cells at the last stage of the lytic cycle^1^. Thus, the bacteriophages have developed several strategies degrading peptidoglycan layers (PGs) which are a major component of bacterial cell walls. For instance, a single lysis gene *E* of bacteriophage ΦX-174 inhibits murein biosynthesis, and then oligomerizes proteinaceous channels in the cell envelope^2^. Particularly, the capacity of protein E to effectively inactivate Gram-negative bacteria led to generate genetically inactivated vaccine constructs known as bacterial ghosts (BGs)^3^,^4^,^5^. BGs are empty cell envelopes of Gram-negative bacteria, which have excellent adjuvant and vaccine delivery system properties. BG generated by ΦX174 lysis gene *E* preserves an intact cell envelope structures containing the potential pathogenic trait of the bacteria, which have the capacity to induce local immunities^6^. However, safety in BG vaccine candidates is still not fully guaranteed due to failure in complete inactivation of target bacterial cells mediated by gene *E*. Haidinger *et al*. cytometrically observed that approximately 1.2% of total *Escherichia coli* cells failed to be inactivated during BG preparation^7^. *Salmonella* BGs inactivated by protein E controlled under the dual promoter system also contained 3 × 10^3^CFU of reproductive cells after 48 hr of lysis^8^. Therefore, several studies attempted to improve the lytic capacity of gene *E* by fusion with other lethal genes relevant to bacteriolysis such as staphylococcal nuclease A gene^9^,^10^. However, the approaches raised the questions whether the BGs produced by the fusion proteins retain the potential to act as potent candidate vaccines. In the present study, we have devised a novel strategy which has not only increased the lysis efficiency of gene *E* but also the immunogenicity of formed BGs. The current study employed holin-endolysin lysis gene cassette originated from bacteriophage λ along with the gene *E* of bacteriophage PhiX174 to generate efficient production of BGs. The holin-endolysin system is composed of *S, R,* and *Rz/Rz1* genes, which encodes holins, endolysins and accessory proteins, respectively, involved in bacterial cell membrane destabilization^11^. In contrast to the lysis gene *E* that interrupts synthesis of the cell membrane compartments, the expression of endolysins is initiated according to the correctly programmed lysis mechanism governed by holin, a small hydrophobic protein, which forms oligomeric pores in the host cytoplasmic membrane at a genetically predetermined time^11^. Consecutively, the endolysins accumulated in cytoplasm is released through the inner membrane pores and then reach to the bacterial cell wall where they hydrolyzes PGs^11^. In this work, *R* gene cassette encoding the holin-endolysin system was integrated with the PhiX174 lysis gene *E* to improve the current BG vaccine platform. The lysis genes were stringently controlled by a convergent promoter system containing a sense λpR promoter with repressor cI857 and an anti-sense ParaBAD promoter with the araC regulatory system^8^. The λpR promoter with the thermolabile repressor cI857 suppresses the lysis gene transcription under 30 °C for the normal growth of the bacterial cells. However, the λpR promoter system may be leaky leading to undesired expression of the lysis genes^12^. To avoid the leaky transcription, in this convergent promoter system an anti-sense RNA of the lysis genes produced by the ParaBAD promoter in the presence of L-arabinose binds to its complementary sense RNA of the lysis gene caused by the leaky λpR promoter^13^,^14^. Consequently, simultaneous activations of the convergent promoters in the ghost plasmid can effectively prevent the translation of mRNA encoding the lysis genes, and the subsequent expression of the lysis genes during the cell growth. The BGs prepared by this novel method, designated as JOL1954, were evaluated for their immunogenicity potential both *in vitro* and *in vivo*.

Here we report the construction of a novel gost cassette lysis system encdoing the gene *E* and the holin-endolysin proteins that enable complete lysis of the Gram-negative bacteria, *Salmonella* Typhimurium. Our results demonstrate that the holin-endolysin and the gene *E* act synergistically to induce efficient and quick bacterial lysis, and preserve the native envelopes structures of bacteria as demonstrated by immunogold transmission electron micrsoscopy. We also observed that ghosts mediated by this novel method drive efficient maturation of murine DCs as evidenced by higher expressions of costimulatory molecules and cytokine gene inductions. Immunization with JOL1954 ghosts elicited efficient humoral and cell mediated immune responses compared to JOL1754 ghosts mediated by gene *E* only. Consequently, protection upon live *Salmonella* challenge was higher in JOL1954 immunized mice compared to JOL1754 vaccinated mice. Taken togather, we conclude that this novel method of preparing BGs is higly efficient and improves immumoregenicity of BGsboth *in vitro* and *in vivo*.

## Results

### Construction of a novel ghost plasmid co-expressing genes *E, R* and *S* in tandem regulated by a convergent promoter system

**Figure 1:**
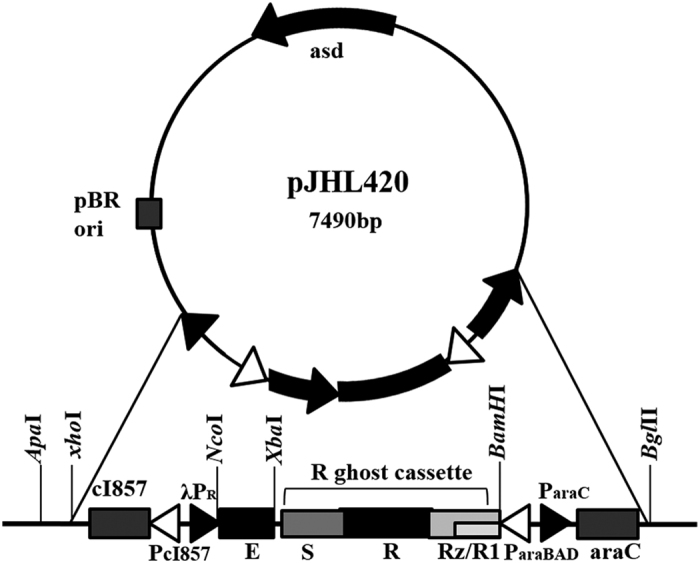
Components of the ghost plasmid pJHL420. The *asd*^+^ plasmid with pBRori carrying constitutive expression system of the lysis genes are regulated by the convergent promoter elements.

**Figure 2:**
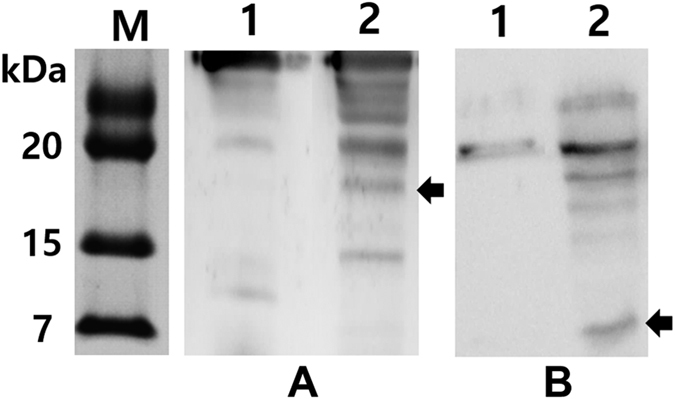
The holin-endolysin or protein E expressed in JOL1954 confirmed by (**A**) rabbit anti-endolysin polyclonal antibody or, (**B**) rabbit anti-protein E polyclonal antibody. The black arrows indicate the expected size of endolysin and protein E, respectively. Lane M, size marker; lane 1, JOL1954 grown in the presence of L-arabinose at 27 °C; lane 2, JOL1954 ghosts produced under the lysis condition.

The ghost cassette developed in this study was comprised of bacteriophage PhiX174 lytic gene *E* and the *R* lysis gene cassette encoding the phage lambda derived holin-endolysin proteins. The R lysis gene cassette were fused in frame to the site between 5′ upstream anti-sense P_araBAD_ promoter and the 3′ downstream regions of the *E* gene and λpR-cI857 promoters in the pJHL420 plasmid (Fig. 1). Thus, the R gene cassette was sequentially expressed with the gene *E* by the thermal and arabinose related induction signal. Co-expression of *E* and *R* genes of pJHL420 plasmid harbored in JOL1954 bacteria were validated by SDS-PAGE and immunoblotting (Fig. 2). By the SDS–PAGE analysis, the expected size of protein E (~8.8 kDa) and endolysins (~17.76 kDa) were observed in JOL1954 lysed bacteria under the condition where arabinose was removed and the temperature was upshifted to 42 °C. Further, the specificity of the lysis inducible proteins was authenticated by the immunoblotting assay using polyclonal hyper-immune sera. Upon Western blot analysis, the protein E and the holin-endolysin showed distinct immunoreactive bands which were absent in the control samples, thus confirmed the expressions of both the proteins (Fig. 2A and B; lane 2). However, no immunoreactive bands were found under the condition repressing the lysis gene expression (Fig. 2A and B; lane 1). These results, thus, indicated that the expression of lysis genes were stringently governed by the convergent promoter elements and R ghost cassette may be cooperatively expressed with the gene *E* to inactivate the target bacteria.

### Characterization of the *Salmonella* ghosts mediated by endolysin and protein E

**Figure 3:**
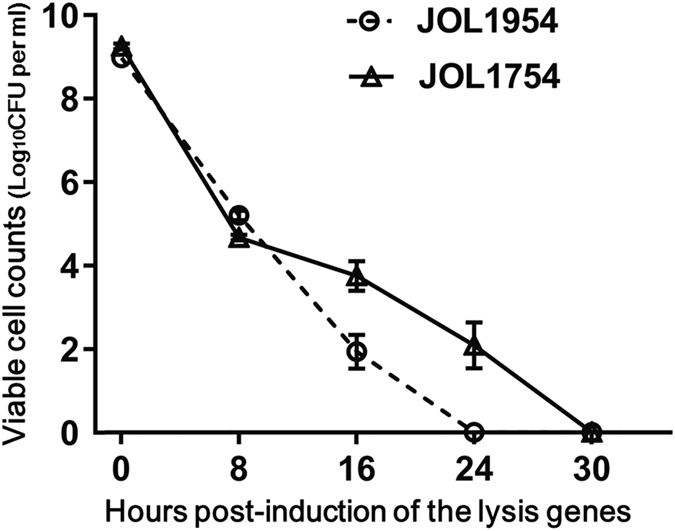
Lysis patterns of JOL1954 and JOL1754 ghost cells. The ghost cells grown up to exponential phase were inactivated by the induction of the lysis genes at 42 °C. The CFU counts were transformed to log base 10 values. The data presented are the mean ± s.d. of three samples.

**Figure 4:**
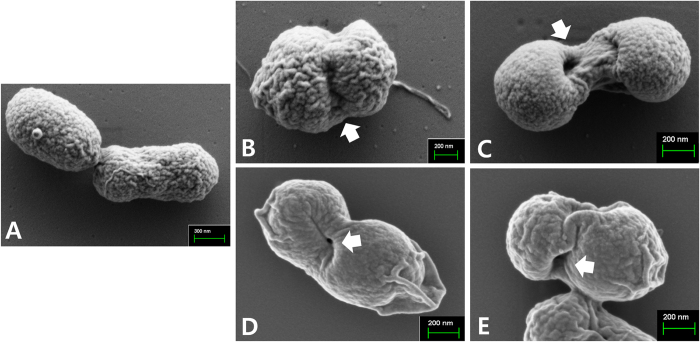
Characterization of JOL1954 *S*. Typhimurium ghost cells by scanning electron microscopy (SEM). (**A**) Intact JOL1954 cells before the lysis. (**B**) JOL1954 ghost cells after 12 hr of the lysis. (**C**) JOL1954 ghost cells after 24 hr of the lysis (**D**) JOL1754 ghost cells after 12 hr of the lysis (**E**) JOL1754 ghost cells after 30 hr of the lysis. The arrows indicate the lysis transmembrane tunnels.

**Figure 5:**
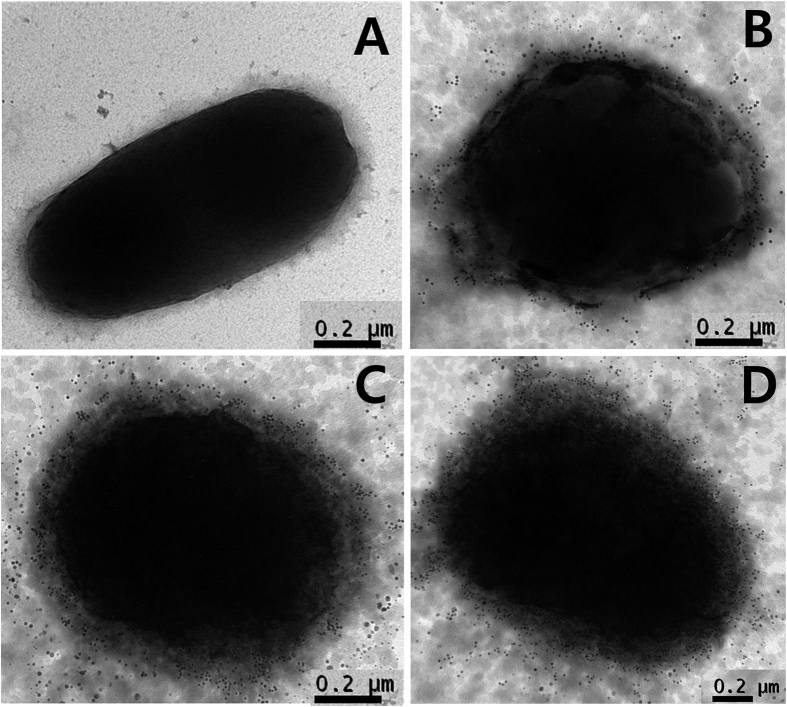
Immunogold labeling of *S*. Typhimurium inactivated by either chemical treatment or the combined lysis gene expression. The bacteria cell was immunogold labeled and negatively stained for transmission electron microscopy. JOL1311 untreated (**A**), JOL1311 treated with 0.2% formalin (**B**), JOL1754 (**C**) and JOL1954 inactivated by the sequential expression of lysis gene E and holin-endolysins (**D**).

To assess whether the concerted expression of the lysis gene *E* and the *R* ghost cassette affect the inactivation efficacy of the *Salmonella* ghost, the lysis pattern of JOL1954 was compared with that of JOL1754. The JOL1954 and JOL1754 were grown in LB broth supplemented with 0.2% L-arabinose at 27 °C overnight up to mid-log phase, which implied that leaky expression of the lysis gene rarely occurred during the growth phase. During the lysis procedure, the number of JOL1954 viable cells was declined faster than those of JOL1754 as evidenced by the lower number of CFU observed in JOL1954 ghost preparation (Fig. 3). No single living cell was detected in the JOL1954 ghost culture after 24 hr of the lysis process, whereas JOL1754 ghost mediated by the sole expression of gene *E* completely lysed after 30 hr. These results supported the conclusion that concomitant production of endolysin in JOL1954 may facilitate the rapid inactivation of the *Salmonella* ghost compared to the lysis behavior of JOL1754. Further, the transmembrane tunnel generated on the surface of JOL1954 ghost was identified by the scanning electron microscopy (SEM) techniques (Fig. 4). The lysis event initiated from middle section of the cells as exhibited by indentation of the bacterial cell (Fig. 4B). After 24 hr of the lysis, the visible pores appeared on the cell surface which was partially collapsed by releasing cytoplasmic contents (Fig. 4C). Further, immunogold labelling showed higher density of immunogold particles along the surface of the JOL1954 ghosts compared to those of formalin-inactivated JOL1311 (Fig. 5). This finding suggests that gene *E* and endolysin inactivation of salmonella bacteria preserves the antigenic traits to a greater extent than those of chemically inactivated bacteria.

### JOL1954 elicited efficient systemic and mucosal antibody responses

**Figure 6:**
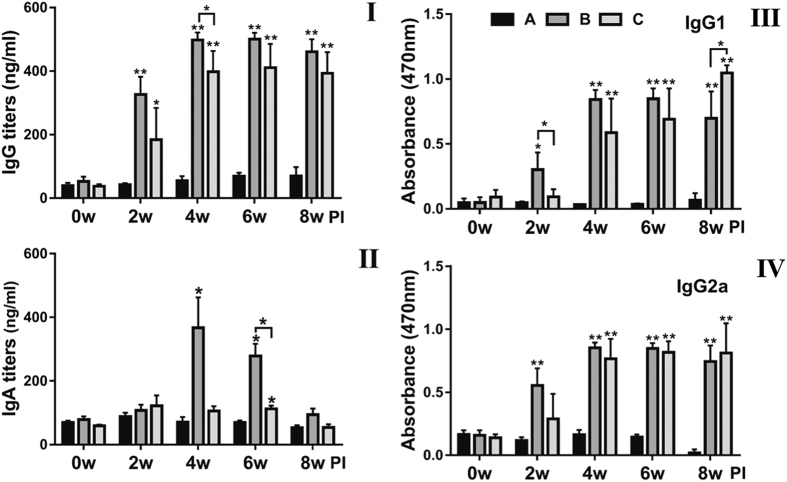
The titers of serum IgG (I), secretory IgA (II), IgG1 (III) and IgG2a (IV) against the *Salmonella*OMP in the immunized mice by ELISA analysis. Secretory IgA was measured in vaginal washes of the mice. Group A, negative controls; group B, the mice immunized with JOL1954 ghost cells; group C, the mice immunized with JOL1754 ghost cells. Bars indicate the mean of all mice (n = 10) in each group and the vertical lines show the standard deviation (s.d.). PI; post-immunization, *P < 0.05, **P < 0.001 (vs. control group A).

The titers of total IgG, isotype specific IgG1 and IgG2a, and secretory IgA (sIgA) elicited in the mice immunized with JOL1954 and JOL1754 ghosts, respectively, were observed by week 8 post immunization (pi) (i.e. week 6pbi). The concentration of IgG detected in mice immunized with JOL1754 and JOL1954, respectively, were markedly raised at week 2, 4, 6 and 8pi (*P* < 0.05) compared to those in the control group (Fig. 6I). At week 4 pi, the titer of IgG in group B was significantly higher than those in JOL1754 (*P* < 0.05). In addition, the level of IgA in group B was significantly increased in comparison with those in group C and control group at week 6pi (Fig. 6I and II). This indicates that *S*. Typhimurium ghosts inactivated by sequential expression of the protein E and endolysinspromoted efficienthumoral immunogenicity compared to those of mice immunized with JOL1754 ghosts lysed by protein E only. As shown in (Fig. 6III & IV), immunization with either JOL1954 or JOL1754 ghost inducedsignificantly enhanced titers of IgG1 and IgG2a, which is an indicator of Th2 and Th1 cell development, respectively,indicating that *Salmonella* ghosts induced mixed type of immune response.

### JOL1954 drive efficient maturation of primary mice BMDCs

**Figure 7:**
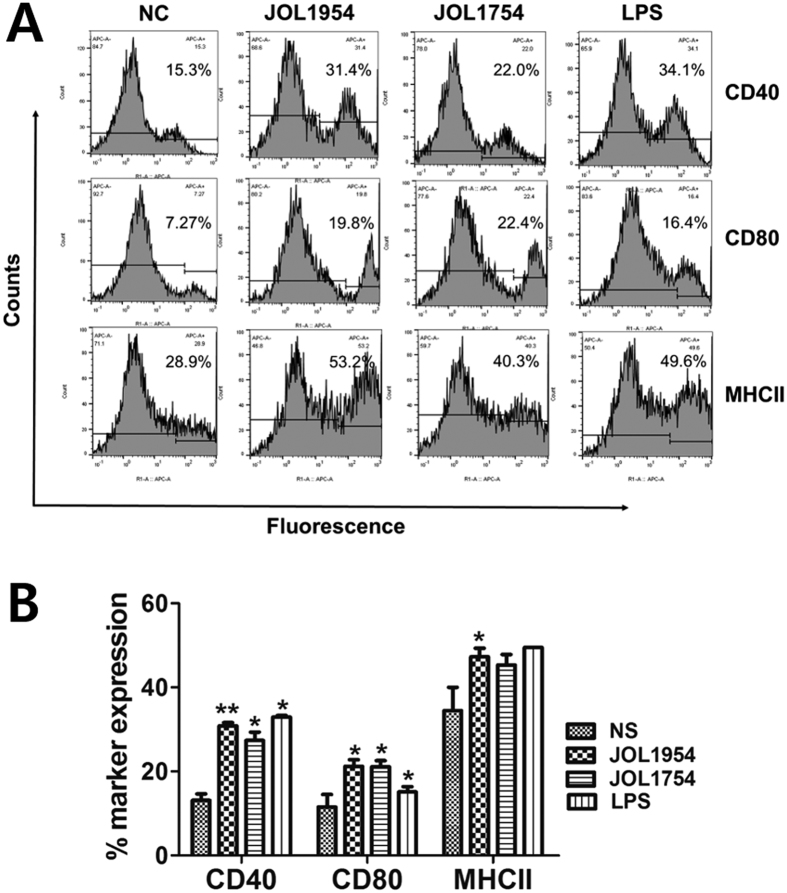
Phenotypic characterization of BMDCs stimulated with either JOL1954 or JOL1754 or LPS as positive control by FACS analysis (**A**) Representative FACS histogram of the surface marker positive cell population. (**B**) Percentages of costimulatory molecule-positive DCs. *P < 0.05, **P < 0.001 (vs. non-stimulated DCs).

**Figure 8:**
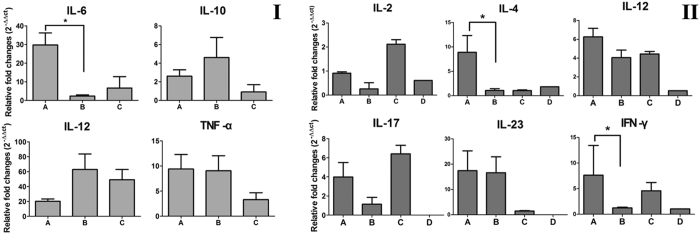
Cytokine mRNA upregulated in DCs co-cultured with the BGs (I) and in naïve CD4^+^ co-incubated with DCs primed with BGs (II). (I) A, DCs primed with JOL1954 ghost; B, DCs primed with JOL1754 ghost; C, non-stimulated DCs. (II) A, CD4^+^ T cell stimulated with DCs primed with JOL1954; B, CD4^+^ T cell stimulated with DCs primed with JOL1754; C, CD4^+^ T cell stimulated with naïve DCs; D, unstimulated CD4^+^ T cell. Relative fold changes were calculated based on 2^−ΔΔCT^ method. *P < 0.05.

The ability of BGs to stimulate naïve murine BMDCs was analyzed in the context of expression of the costimulatory molecules and induction of immuno-regulatory cytokines. Uptake of either JOL1754 or JOL1954 ghosts by the *in vitro* differentiated DCs significantly increased surface expression of co-stimulatory molecules, MHC-II, CD40, and CD80. DCs stimulated with JOL1954 ghosts markedly raised expression of MHC II molecules compared to that of JOL1754 ghosts (*P* < 0.05) while similar levels of CD40 and CD80 were observed (Fig. 7). In addition, the cytokine genes in the DCs were significantly upregulated in response to the uptake of BGs. Particularly, the increased mRNA expression of IL-12, indicating full maturation of DCs, was observed in the DCs co-cultured with either JOL1754 or JOL1954 ghosts, whereas IL-6 cytokine, polarizing Th0 into Th17 cells, was significantly upregulated (29.8 ± 12.9-fold increase) in the DC primed with JOL1954 ghosts compared to those in DCs co-cultured with JOL1754 (Fig. 8I). The cytokine IL-6 helps in the development of effective and potent mucosal immune responses, and protects the host against bacterial and viral infections^15^,^16^. This result might explain why JOL1954 ghost immunization induced significantly (*P* < 0.05) higher mucosal IgA responses in mice (Fig. 6II) and effective immune protection against the lethal bacterial challenge as observed in this study. The mRNA inductions of TNF-α and IL-10 were comparable in DCs stimulated with either JOL1754 or JOL1954 ghosts (Fig. 8I).

To further examine the issue that the DCs co-incubated with the BGs can present the foreign antigens to naïve CD4^+^ T cell *in vitro*, we assessed the *in vitro* priming ability of the DCs. The immunomodulatory cytokine mRNA expressions containing IL-12, IL-17 and IL-23 were significantly enhanced in the CD4^+^ T cells stimulated with either JOL1754 or JOL1954 ghosts (Fig. 8II). However, mRNA expression of the cytokines, IL-4 and IFN-γ, representing Th2 and Th1 arms of immunity, increased 8.9 ± 6.0 and 7.6 ± 10.0 fold, respectively, in CD4^+^ T cell co-cultured with DCs primed with JOL1954, while the cytokine mRNA rarely expressed in CD4^+^ T cells co-incubated with the DCs stimulated with JOL1754 ghosts (1.2 ± 0.2 and 1.07 ± 0.6-fold increase for IL-4 and IFN-γ, respectively) (Fig. 8II). The mRNA induction levels of IFN-γ and IL-10 were comparable, indicating the mixed type of Th1 and Th2 immune responses elicited by JOL1957 ghosts. These results clearly demonstrate that JOL1954 ghosts efficiently stimulate naïve CD4 T cells and induce cytokine profile of both Th1 and Th2 type of immunity.

### JOL1954 induce efficient CM1 responses

**Figure 9:**
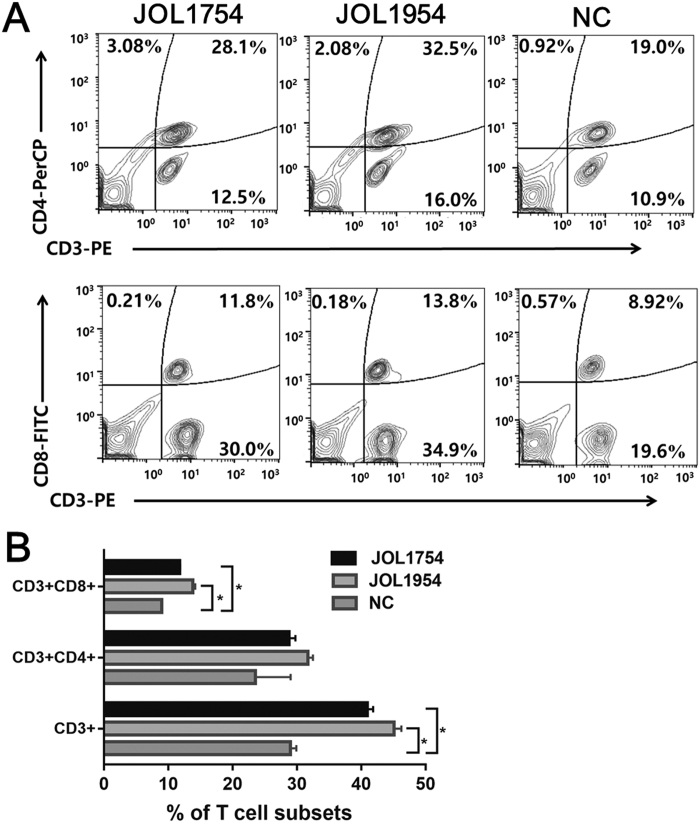
Flow cytometric analysis for CD3^+^CD4^+^ splenic T lymphocyte population. (**A**) Representative flow cytometry scatter dot plots for CD3^+^, CD3^+^CD4^+^, CD3^+^CD8^+^ splenic T cell populations of immunized mice (n = 5) and the negative controls (NC) (n = 5). The subpopulations are expressed as a percentage of the gated cells. II) CD3^+^, CD3^+^CD4^+^ and CD3^+^CD8^+^ splenic T-cell subsets in immunized mice and NC. The data are presented as the mean ± s.d. *P < 0.05 when the values are compared with the negative control group.

The alteration of T cell population following the immunization was evaluated by FACS analysis in the splenocytes isolated from the immunized mice. The proportion of CD3^+^ total T cell subset increased averagely 10.3 and 15.2% in the mice immunized with JOL1754 and JOL1954, respectively, compared to those of non-immunized mice (Fig. 9A and B). The fraction of cytotoxic CD3^+^CD8^+^ T cells, which is necessary for immune defense against intracellular pathogens^17^ were significantly augmented in the immunized mice compared to those of the control. The JOL1954 ghost enhanced the differentiation of naïve T cell population at a level similar to that achieved by JOL1754 ghost (Fig. 9B), suggesting that both JOL1754 and JOL1954 ghosts have potential to elicit antigen-specific T cell responses in immunized mice.

### JOL1954 protected mice against lethal *Salmonella* challenge

**Figure 10:**
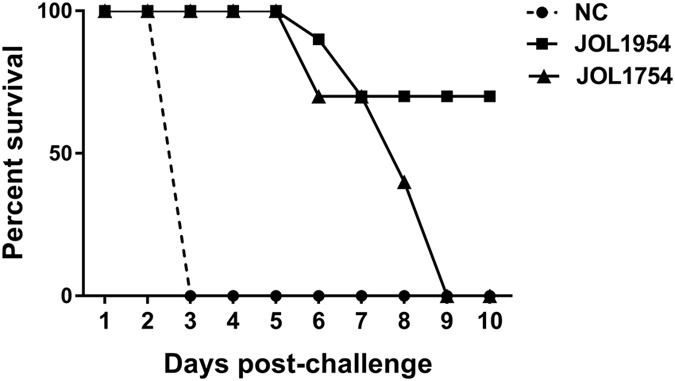
Protective efficacy of JOL1954 and 1954 BG vaccination. A lethal challenge against a virulent *S*. Typhimurium, strain JOL401, was performed in the immunized mice, and survival rate of the mice immunized (n = 10) with either JOL1954 or JOL1754 or PBS was recorded.

To assess the protective efficacy of the *Salmonella* strain carrying the newly constructed ghost plasmid against *S*. Typhimurium infection, groups of intramuscularly immunized mice were challenged with a lethal dose of a virulent *S.* Typhimurium, strainJOL401. All mice in the control group and immunized with JOL1754 died by day 3 and day 9post-infection, respectively, while 70% of the mice immunized with JOL1954 survived by the end of observation period (Fig. 10). The obtained result further suggest that JOL1954 ghost generated by concerted expression of two lysis genes have improved immunogenicity and thus provided robust protection against the lethal challenge compared to JOL1754 ghosts.

## Discussion

In the work presented herein, we demonstrated for the first time that simultaneous utilization of phage lambda endolysin and bacteriophage ΦX174 protein E substantially contributed to the development of desired BGs. We observed that the co-expression of two lysis genes allowed rapid and highly efficient inactivation rate of *S*. Typhimurium during the ghost formation procedure. Additionally, the immunization with the derived *Salmonella* ghost strain elicited efficient humoral and cell mediated immune responses, and improved protection efficacy against a lethal challenge in the mice. The morphological alteration in JOL1954 ghosts inactivated by the underlying lysis mechanism was identified by SEM study. The pores originating from the lysis gene expressions were distinctly visible on the collapsed body with surface folds in the JOL1954 ghosts. The changes supported that sequential expression of the lysis genes in the bacteria was successfully accomplished. Further, immunogold labelling study demonstrated that the antigenic surface structures of JOL1954 ghosts were not significantly degraded during the lysis procedure, resulting in no alteration of their antibody binding specificity.

The gene *E* codes for a very small protein which is 91 amino acids in length that contain hydrophobic moieties within its N-terminal region. When expressed, protein E is inserted into the inner and outer membrane of gram-negative bacteria by proton motive force, resulting in the approximate formation of the transmembrane tunnel structures of 40–80 nm in diameter^18^. This tunnel like structure spans the inner and outer membrane of bacterial cells through which cytoplasmic contents including DNA are expelled out, leaving behind empty cell envelopes known as BGs. On the other hand, endolysins are accumulated in the cytoplasm until holin, a small hydrophobic protein, generates the inner membrane pores. At the time when holin determined, endolysins reach the PG layer through the pore and hydrolyze the bacterial cell wall^19^. In this study, potent lytic activity exerted by sequential transcription of two lysis genes resulted in rapid and efficient production of JOL1954 ghost in approximately 24 hr (Fig. 3). In the previous studies, a complete reduction of viable *Salmonella* cells induced by the gene *E* only was achieved in 4 hr of lysis on average^8^,^20^,^21^, which imply that R ghost cassette apparently rendered the preparation of BGs effective. The sole use of endolysin to lyse gram-negative bacteria has been limited due to a lack of its capacity to cross the bacterial outer membrane (OM) despite its robust PG hydrolytic activity^22^. Thus, the previous study developed the recombinant endolysin combining the protein such as a Artilysins peptide which assisted in the transportation of endolysin to the OM^23^. In this context, based on the results presented, we presumed that the expressed endolysins could gain access to PGs through transmembrane channel generated by the protein E, not the inner membrane pores induced by the holin protein as originally programmed. The lysis ofJOL1954 cells could be exerted by the PG layers hydrolyzed by endolysin, resulting in rapid formation of the ghost cells. Collectively, *R* gene encoding endolysin introduced into plasmid pJHL420 was responsible for the efficient production of *S*. Typhimurium ghost cells. Previously, we and others show that killing in *E. coli* is never absolute and some rare non-lysed reproductive cells are found within the BG preparation^4^,^9^. The complete killing and genomic inactivation of *E. coli* has been achieved by the expression of staphylococcal nuclease, *SNUC* gene, along with the gene *E*-mediated lysis as demonstrated by Haidinger *et al*.^9^ The *SNUC* gene is a phosphodiesterase which cleaves single or double stranded DNA or RNA into dinucleotides or nucleosides, and its action is dependent on Ca^++^ and Mg^++^ ions. Along with the gene *E*, other immunomodulatory genes have been expressed on the surface of BGs, but complete killing of the bacteria was never absolute^24^,^25^. In the present study, the expression of holin-endolysin along with the gene *E* has not only resulted in the complete killing of the *Salmonella* bacteria, but has concurrently increased the immunogenicity of the JOL1954 BGs.

Antibodies generated by B cells recognize epitopes of intact antigens in its native form and are essential effectors that regulate protection against *Salmonella* infection^26^. In this study, the significantly elevated titers of IgG and sIgA were detected in the mice immunized with JOL1954 ghosts compared to those of mice injected with JOL1754 (Fig. 6). Particularly, the level of sIgA, which is elementary for mucosal immunity, was significantly enhanced at week 2 and 4 pbi with JOL1954. These results indicated that the surface antigenic determinants of JOL1954 ghost cells did not appear to be altered compared to those of JOL1754 ghosts. Further, high immunogold labeling density displayed to the surface of JOL1954 ghost indicated that possible alteration of the antigenic properties of JOL1954 ghost by the lysis procedure did not occur. BGs mediate active immunization against their own envelope structural components and bias the immune response either Th1 or Th2 depending upon the presentation of antigen to antigen presenting cells^27^. The activation of DC, one of the potent antigen-presenting cells, is initiated by recognition of pathogen associated molecular patterns (PAMPs) from pathogens by innate immune receptors, for instance, TLRs. The expression of MHC-peptide complex, costimulatory molecules and subsequent production of immunomodulatory cytokines from the activated DCs direct the differentiation of the naïve T cells into effector T cells, and subsequently induce adaptive immunity^28^. In this study, a significant increase of CD40, CD80, and MHC II surface makers expressed on BMDCs were observed in cultures co-incubated with JOL1954 ghosts *in vitro*. Considering a marked proliferation of CD3^+^CD4^+^ and CD3^+^CD8^+^ activated T cells *in vivo* in the mice immunized with JOL1954 ghost, the primed DCs could efficiently present the JOL1954 ghosts to naïve T cell *in vivo*. Concomitantly, increased concentration of IgG1 and IgG2a as indicators of Th2 and Th1 related immune responses, respectively, in the immunized mice implicated that the increased fraction of CD3^+^CD4^+^ T cell was differentiated into effector Th1 and Th2 cells^29^. The markedly upregulated IFN-γ and IL-4 mRNA in CD4^+^ T cell primed with JOL1954 ghost stimulated BMDCs *in vitro* also corroborated the capacity of JOL1954 ghost to stimulate Th1 and Th2 related immunity. The prominent immunogenic capacity of JOL1954 ghosts in the mice, consequently gave rise to high protection efficacy against a lethal dose of a virulent *S*. Typhimurium. Considering that antibody and T cell-mediated immune response are critical for protection against *S*. Typhimurium infection^30^, the antigenic and immunogenic properties of JOL1954 ghosts, thus, seem to be superior to those of JOL1754 ghosts as evidenced by efficient elicitation of antibody responses and protection against lethal challenge. The increased immunogenicity of JOL1954 ghosts could also be attributed to the presence of R ghost cassette proteins as they might have adjuvant activity. The adjuvant potential of R gene cassette, besides role in improving lysis efficiency, thus needs to be validated in future studies.

In conclusion, we show for the first time that the co-expression of PhiX174 lytic gene *E* and lambda phage holin-endolysin induce rapid and efficient production of BGs compared to the gene *E* mediated lysis. Further, we observe that the BGs produced by E + R system displayed more immunogenic potential compared to ghosts generated by the sole expression of gene *E*. We believe that this novel strategy has the potential to generate highly efficient inactivated candidate vaccines that could replace the currently available bacterial vaccines.

## Materials and Methods

### Bacterial strains, plasmids and growth media

Bacterial strains and plasmids used in this study are listed in Table 1. Theaspartate-semialdehyde dehydrogenase (*asd*) gene deleted bacterial mutants were grown in LB medium supplemented with diaminopimelic acid (DAP; 50 μg/ml). The bacterial strain carrying the ghost lysis plasmid pJHL172 or pJHL420were cultivated at 27 °C in LB medium supplemented with 0.2% L-arabinose. All bacterial strains were kept at −80 °C until further use.

### Construction of a novel plasmid harboring the *S* and *R* lysis gene cassette

The lysis gene cassette encoding holin and endolysin of bacteriophage λ was chemically synthesized (Bioneer, South Korea), which was referred to as ‘R ghost cassette’. The cassette consisted of the open reading frames (ORFs) of *S* and *R* genes, and an overlapping ORF of Rz/Rz1 gene (Fig. 1). To yield the efficient lysis gene cassette encoding protein E and R ghost cassette, the N-terminus of R ghost cassette was digested with XbaI and BamHI and cloned in frame downstream of the *E* gene sequence of T-easy vector harboring the *E* lysis cassette with a convergent promoter system derived of pJHL172^8^. The resultant plasmid was designated as pJHL350 where the subcloned 1433-bp R ghost cassette was placed between upstream of the arabinose-inducible araBAD promoter regulated by the AraC protein and downstream of *E* gene. The plasmid obtained was transformed into *E. coli* DH5α to minimize instability of the plasmid. Subsequently, the 4.5-kb DNA fragment carrying the constructed lysis gene cassette harbored in pJHL350 was substituted with the 1.7-kb *E* lysis gene cassette in the *asd*^+^ plasmid pJHL172 using BglII and XhoI restriction site. The resulting plasmid, pJHL420, was initially transformed into a Δ*asd E. coli* χ6212 (JOL232) to maintain integrity of the plasmid, and then electrophoreticallyintroduced into Δ*asd S.* Typhimurium, strain JOL1311, and the constructed strain was designated as JOL1954. For comparative study, the pJHL172 plasmid was transformed into JOL1311to produce the *Salmonella* ghost inactivated byan expression of protein E only. For purification ofthe endolysin protein, the *R* gene was amplified by PCR from the plasmid pJHL420 using the following oligonucleotides: R_F: 5′-CCGCGAATTCATGGTAGAAATCAATAATCAACG-3′, R_R: 5′-CCGCCTCGAGTACATCAATCTCTCTGACCG-3′. PCR-amplified products were ligated into the protein expression vector pET28a, and then the resultant plasmid was finally introduced into competent *E. coli* BL21 (DE3) strain for protein expression. The N-terminally 6×-His-tagged endolysin expressed in *E. coli* BL21 was purified following the protocol previously described^25^. The concentration of purified protein was quantified using Bradford reagent (Bio-Rad Laboratories, Hercules, CA), dialysed against PBS, filtered, and stored at −20 °C until use.

### Characterization of the *Salmonella* ghosts media×ted by endolysins and gene E

The expressions of the protein E and endolysin in JOL1954 ghost strain were validated by Western blot analysis using hyperimmune rabbit serum against the protein E or endolysinaccording to the previously published protocol^8^. The morphological alteration of the lysed JOL1954 and JOL1754 was visualized by using scanning electron microscopy (SEM) with a 12 hr interval during the lysis procedure as previously described^8^. In order to further elucidate whether the lysis mediated by the combined gene expression alter the native surface antigenic structures of JOL1954 and JOL1754, immunogold labeling transmission electron microscopy was performed following the protocol previously published. Briefly, JOL1954 ghost cells were fixed at 4 °C overnight with 2% paraformaldehyde and 2% glutaraldehyde. Carbon formvar-coated copper grids (200-meshes) were placed on the cells suspended in PBS for 10 min. After washing twice with PBS, the cells bound to the grid were blocked in 2% BSA for 30 min. Subsequently, the grids were washed and incubated with a chicken polyclonal antibody raised against JOL1311 strain (1:300) as a primary antibody for 1 hr followed by incubation with 10 nm gold labeled goat anti-chicken IgY(1:100; Abcam Inc., USA) used as a secondary antibody for 30 mins. Finally, the cells on the grids were stained with 2% uranyl acetate and viewed. To compare immunoreactivity of JOL1954 ghosts with the cells inactivated by chemical treatment, JOL1311 strain inactivated by 0.2% formalin at 37 °C for 2 hr was prepared and processed following the protocol above.

### Lysis efficiency

To generate *S*. Typhimurium ghost, a single colony of JOL1954 was grown to mid-log phase with a slow agitation (80 rpm) in LB broth containing 0.2% L-arabinose at 27 °C. The cultures were harvested by centrifugation to remove the remaining L-arabinose, and the cells resuspended with LB broth were incubated at 42 °C with 150 rpm agitation. The lysis efficiency of the *S*. Typhimurium ghost was determined by cell viability of the culture periodically collected. The cell viability was monitored by counting the number of colony forming units (CFU) as described previously^4^. To assess the lytic capacity of endolysins incorporated with the *E* lysis gene cassette, the magnitude of JOL1954 ghosts induced under the optimal lysis condition was compared to that ofJOL1754 ghosts by protein E only, at the various time intervals. At the end of lysis procedure, the cells were harvested by centrifugation at 4,000 × g for 20 min and the pellets were stored at −70 °C until further processing.

### Immunization and challenge with S. *Typhimurium*

All animal experimentation work was approved by the Chonbuk National University Animal Ethics Committee (CBNU2015-00085), and was carried out according to the guidelines of the Korean Council on Animal Care and Korean Animal Protection Law, 2007; Article 13 (Experiments with Animals). Female 5-weeks-old BALB/c (n = 45) were randomly divided into three groups. The animals were acclimated for a week before the immunization. Groups A and B (n = 15, each) were intramuscularly immunized with 1 × 10^8^ ghost cells of JOL1954 and JOL1754, respectively, at day 0 and boosted at 14^th^ day post primary immunization. Group C mice were injected with sterile PBS as a non-immunized control. At week 0, 2, 4, 6 and 8 post immunization (pi), sera were isolated from blood obtained via orbital sinus, and vaginal washes were collected by washing with 100 μl of sterile PBS. At week 6 pbi, the mice in all groups were intraperitoneally challenged with a lethal dose (1 × 10^6^ cells) of a wild-type of *S.* Typhimurium.

### Antigen specific ELISA

The titers of total immunoglobulin (Ig) G, G1, G2a and IgA against *S*. Typhimurium outer membrane protein (OMP) were analyzed in the immunized and non-immunized mice by ELISA^31^. The OMP protein was extracted from the wild-type *S*. Typhimurium, JOL401 strain, according to the previously reported method^32^. The final concentration of serum IgG and secretory IgA were analyzed by a standard curve of purified mouse immunoglobulins (Southern Biotechnology, Birmingham, AL). The end point titers of IgG isotypes, IgG1 and IgG2a, in sera were determined as previously described^33^.

### Dendritic cell analysis

To assess T-cell related immune responses depending on how the BGs stimulate naïve DCs *in vitro*, the expressions of costimulatory molecules were observed in murine bone-marrow derived dendritic cells (BMDCs) stimulated with either JOL1754 or JOL1954 BGs using flow cytometry. In addition, upregulation of cytokine genes expressed in the primed DC, which is necessary for the ensuing immune responses, was confirmed by quantitative real-time PCR assay (qRT-PCR). Further, the capacity of activated DCs to stimulate autologous CD4^+^ T cells *in vitro* was measured by expression of immunomodulatory T- cell cytokine transcripts via qRT-PCR. BMDCs were aseptically isolated from female C57BL/6 mice at 6 week of age (n = 3) and harvested following the protocol previously described with slight modifications^34^. Briefly, the 1 × 10^6^cells cultured in RPMI medium supplemented with 10% FBS, penicillin (10 units/ml), streptomycin (10 μg/ml), L-glutamine (0.29 mg/ml) and cytokines (10 ng/ml murine GM–CSF and 5ng/ml IL-4). On day 5 of culture, proliferating BMDCs loosely adherent to the bottom of the plate were collected, washed and seeded in the 6 well cell culture plates. Subsequently, the cell population was treated with either BGs (10 particles/cell) or LPS (500 ng/ml, serotype O127:B8, Sigma-Aldrich, St. Louis, MO) as a positive control for 24 hr. The expression of surface markers on the primed BMDCs was confirmed by performing FACS analysis as previously described^35^, following labeling of cells with FITC anti-mouse CD11c, APC anti-mouse CD40, APC anti-mouse CD80 and APC anti-mouse MHC class II (all MiltenyiBiotec). The proportion of the cells expressing the surface markers of the costimulatory molecules were estimated in CD11c-positive gated DCs. The upregulation of cytokine genes in the primed BMDCs containing IL-6, IL-10, IL-12, and TNF-α were detected by qRT-PCR assay^35^. To characterize the interaction of naïve CD4^+^ T cell with the primed BMDCs, autologous CD4^+^ T cells were collected from splenocytes in female C57BL/6 mice at 6 week of age (n = 4) using a magnetic bead-based CD4^+^ T cell isolation kit (Miltenyi Biotec) following the manufacturer’s instruction. The 1 × 10^8^ of the isolated CD4^+^ T cells cultured in RPMI medium were co-incubated with the 1 × 10^6^ of the primed BMDCs for 24hr at 37 °C in a humidified 5% CO_2_ incubator. The expression level of cytokine mRNA containing IL-2, IL-4,IL-12, IL-17,IL-23, and IFN-γ was assessed in the differentiated CD4^+^ T cells by using qRT-PCR assay^35^. All the primer pairs applied in the cytokine assay were acquired from the previous studies^36^,^37^.

### T cell responses elicited by *Salmonella* ghost harboring the new ghost plasmid

Following the immunization, the magnitude of differentiation of splenic T lymphocytes was evaluated by using FACS analysis. At week 1 post immunization, splenocytes were aseptically isolated from five mice of each group. The prepared splenocytes were washed in PBS and RPMI1640 medium, and seeded in 96 well cell culture plate at the density of 5 × 10^5^ cells per well. After washing with FACS buffer (MiltenyiBiotec, Bergisch Gladbach, Germany), the cells were stained with anti-CD3-PE, CD4-perCP-vio700 and CD8-FITC antibodies (MiltenyiBiotec). The percentage of CD4^+^ or CD8^+^ population was estimated in the CD3^+^ gated cells.

### Statistical analysis

The difference between the immunized and the control groups was determined by the mean of independent-samples *t*-test using GraphPad Prism version 7.0 (GraphPad Software, San Diego, CA). The FACS data were analyzed using FlowJo software (Treestar, Inc., San Carlos, CA). A *P*-value less than 0.05 were considered statistically significant.

## Additional Information

**How to cite this article**: Won, G. *et al*. Improved lysis efficiency and immunogenicity of *Salmonella* ghosts mediated by co-expression of λ phage holin-endolysin and ΦX174 gene E. *Sci. Rep.* **7**, 45139; doi: 10.1038/srep45139 (2017).

**Publisher's note:** Springer Nature remains neutral with regard to jurisdictional claims in published maps and institutional affiliations.

## Figures and Tables

**Table 1:** Bacterial strains and plasmids utilized in this study.

Strain/plasmids	Description	References
*E. coli*		
DH5α	*fhuA2 Δ(argF-lacZ)U169 phoA glnV44 Φ80 Δ(lacZ)M15 gyrA96 recA1 relA1 endA1 thi-1 hsdR17*	Lab stock
BL21(DE3)	139(*ara-leu*)7697 *gal*U*gal*K*rps*L (Str^r^)*end*A1 *nup*GF^−^*ompThsdSB*(rB^−^mB^−^)*dcmgalλ*(DE3) pLysSCmr	Lab stock
χ6212	F-λ-φ80 Δ(*lacZYA*-*argF) endA1 recA1 hadR17 deoR thi-1 glnV44 gyrA96 relA1 ΔasdA4*	Lab stock
*Salmonella* Typhimurium		
JOL401	Wild typeisolate from chicken	Lab stock
JOL990	Wild typeisolate from porcine, challenge strain	Lab stock
JOL1311	*Δasd*, used as base vaccine strain	Lab stock
JOL1754	JOL1311 containing pJHL172	This study
JOL1954	JOL1311 containing pJHL420	This study
Plasmids		
pJHL319	T-easy vector harboring E gene ghost cassette with the convergent promoter system	This study
pJHL350	T-easy vector harboring *E* gene and the R ghost cassette with the convergent promoter system	This study
pJHL172	asd^+^vector, pBRoriplasmid harboring cI857/λP_R_ promoter, *araC* P_*araBAD*_, *phi*X174 lysis gene *E*	8
pJHL420	asd^+^vector, pBRoriplasmid harboring cI857/λP_R_ promoter, *araC* P_*araBAD*_, *phi*X174 lysis gene *E,* the *R* ghost cassette composed of *S, R and R1/Rz*genes	This study
